# Genetic Evidence for Two Carbon Fixation Pathways (the Calvin-Benson-Bassham Cycle and the Reverse Tricarboxylic Acid Cycle) in Symbiotic and Free-Living Bacteria

**DOI:** 10.1128/mSphere.00394-18

**Published:** 2019-01-02

**Authors:** Maxim Rubin-Blum, Nicole Dubilier, Manuel Kleiner

**Affiliations:** a Max Planck Institute for Marine Microbiology, Bremen, Germany; b Israel Limnology and Oceanography Research, Haifa, Israel; c MARUM, University of Bremen, Bremen, Germany; d Department of Plant and Microbial Biology, North Carolina State University, Raleigh, North Carolina, USA; University of British Columbia

**Keywords:** carbon dioxide assimilation, carbon metabolism, electron transport, lithoautotrophic metabolism, symbiosis

## Abstract

Very few bacteria are able to fix carbon via both the reverse tricarboxylic acid (rTCA) and the Calvin-Benson-Bassham (CBB) cycles, such as symbiotic, sulfur-oxidizing bacteria that are the sole carbon source for the marine tubeworm Riftia pachyptila, the fastest-growing invertebrate. To date, the coexistence of these two carbon fixation pathways had not been found in a cultured bacterium and could thus not be studied in detail. Moreover, it was not clear if these two pathways were encoded in the same symbiont individual, or if two symbiont populations, each with one of the pathways, coexisted within tubeworms. With comparative genomics, we show that Thioflavicoccus mobilis, a cultured, free-living gammaproteobacterial sulfur oxidizer, possesses the genes for both carbon fixation pathways. Here, we also show that both the CBB and rTCA pathways are likely encoded in the genome of the sulfur-oxidizing symbiont of the tubeworm Escarpia laminata from deep-sea asphalt volcanoes in the Gulf of Mexico. Finally, we provide genomic and transcriptomic data suggesting a potential electron flow toward the rTCA cycle carboxylase 2-oxoglutarate:ferredoxin oxidoreductase, via a rare variant of NADH dehydrogenase/heterodisulfide reductase in the E. laminata symbiont. This electron-bifurcating complex, together with NAD(P)^+^ transhydrogenase and Na^+^ translocating Rnf membrane complexes, may improve the efficiency of the rTCA cycle in both the symbiotic and the free-living sulfur oxidizer.

**IMPORTANCE** Primary production on Earth is dependent on autotrophic carbon fixation, which leads to the incorporation of carbon dioxide into biomass. Multiple metabolic pathways have been described for autotrophic carbon fixation, but most autotrophic organisms were assumed to have the genes for only one of these pathways. Our finding of a cultivable bacterium with two carbon fixation pathways in its genome, the rTCA and the CBB cycle, opens the possibility to study the potential benefits of having these two pathways and the interplay between them. Additionally, this will allow the investigation of the unusual and potentially very efficient mechanism of electron flow that could drive the rTCA cycle in these autotrophs. Such studies will deepen our understanding of carbon fixation pathways and could provide new avenues for optimizing carbon fixation in biotechnological applications.

## Abstract (precis)

Primary production on Earth is dependent on autotrophic carbon fixation, which leads to the incorporation of carbon dioxide into biomass. Multiple metabolic pathways have been described for autotrophic carbon fixation, but most autotrophic organisms were assumed to have the genes for only one of these pathways. Our finding of a cultivable bacterium with two carbon fixation pathways in its genome, the rTCA and the CBB cycle, opens the possibility to study the potential benefits of having these two pathways and the interplay between them. Additionally, this will allow the investigation of the unusual and potentially very efficient mechanism of electron flow that could drive the rTCA cycle in these autotrophs. Such studies will deepen our understanding of carbon fixation pathways and could provide new avenues for optimizing carbon fixation in biotechnological applications.

## OBSERVATION

Primary production by autotrophic organisms drives the global carbon cycle. Currently, seven naturally occurring pathways for inorganic carbon fixation are known in autotrophic organisms (1, 2). The dominant carbon fixation pathway used by plants, algae, and many bacteria is the Calvin-Benson-Bassham (CBB) cycle. The six alternative pathways include among others the reverse tricarboxylic acid (rTCA) cycle and the recently discovered reversed oxidative TCA cycle (roTCA) (1, 3, 4). Only a few autotrophic bacteria have more than one carbon fixation pathway (5). These bacteria include a closely related group of sulfur-oxidizing symbionts of marine tubeworms such as *Riftia*, *Escarpia*, *Tevnia*, and *Lamellibrachia*, which have and express both the oxygen-sensitive rTCA and the oxygen-tolerant CBB cycle (6–11). The only known free-living bacteria that may have all the genes for both cycles are the large sulfur bacteria *Beggiatoa* and *Thiomargarita* spp. (12–14). The CBB cycle in the symbionts and the large sulfur bacteria is potentially more energy efficient than the classical version of the CBB cycle based on the replacement of the fructose-1,6-bisphosphatase with a pyrophosphate-dependent enzyme (12, 13, 15, 16). In addition, it is likely that the interplay between the CBB and rTCA cycle under fluctuating redox conditions contributes to the high efficiency of carbon fixation in tubeworm symbioses (6, 7, 17) and consequently to the extremely high growth rates of tubeworms, which grow faster than any other known invertebrate (18).

Given that tubeworm symbionts and large sulfur bacteria could not yet be cultivated, it was not possible to investigate the cooccurrence of their two carbon fixation cycles in detail. In this study, we sequenced a high-quality genome (99.5% completeness as estimated by CheckM) and transcriptome of the symbiont from the tubeworm Escarpia laminata and compared its genome to those of other tubeworm symbionts and free-living microbes. These comparisons revealed the cooccurrence of the complete set of genes for the CBB and rTCA cycles in a cultured bacterium. This discovery will enable future studies of the biochemical and physiological mechanisms that enable the interplay between these two carbon fixation pathways.

### Cooccurrence of rTCA cycle genes with RuBisCO in symbiotic and free-living *Gammaproteobacteria*.

**FIG 1:**
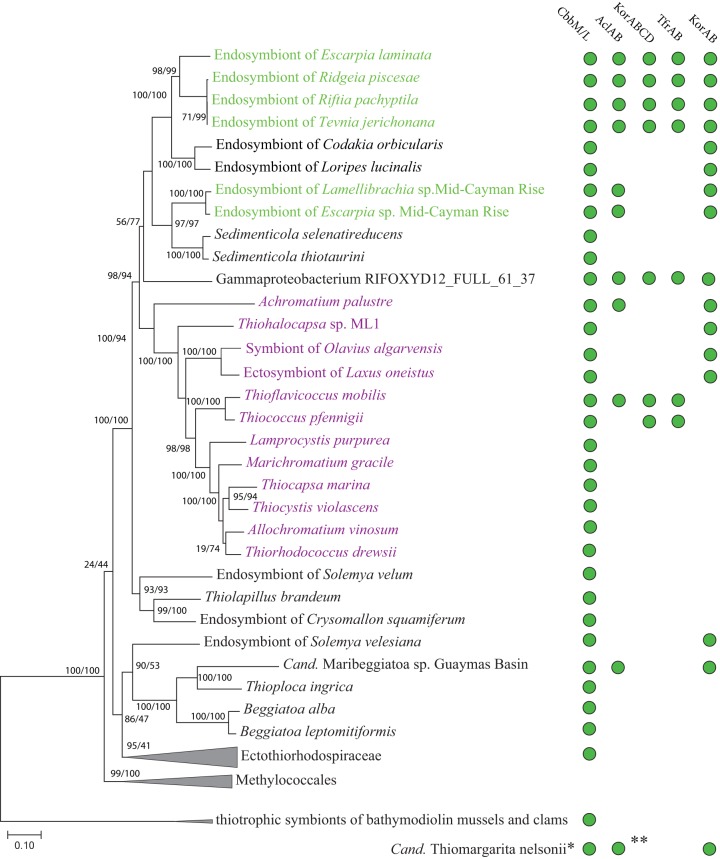
Phylogenomic tree showing occurrence of RuBisCO (CbbM/CbbL), ATP citrate lyase (AclAB), 4-subunit 2-oxoglutarate:ferredoxin oxidoreductase (KorABCD), putative thiol:fumarate reductase (TfrAB), and 2-subunit 2-oxoglutarate:ferredoxin oxidoreductase (KorAB) in the genomes of tubeworm symbionts (green), purple sulfur bacteria (purple), and other related bacteria (58 organisms total, alignment of 2,526 amino acid sites from 23 single-copy markers). The maximum likelihood tree was built with IQ-TREE using the LG+R6 model of substitution. The tree is unrooted, although the outgroup “thiotrophic symbionts of bathymodiolin mussels and clams” is drawn at the root. Branch labels are SH-aLRT support (%)/ultrafast bootstrap support (%). Accession numbers are provided in Table S2. *, was not included in the tree due to several missing single-copy marker genes or multiple versions of these genes, making an accurate phylogenomic placement challenging. **, only the *aclB* gene was present.

Genes for enzymes that are specific to the rTCA pathway, that is, the type II ATP citrate lyase (ACL, *aclAB* genes), 2-oxoglutarate:ferredoxin oxidoreductase (OGOR*, korABCD* genes), and a putative fumarate reductase (*tfrAB* genes, homologs of genes encoding a thiol:fumarate reductase from Methanobacterium thermoautotrophicum [19]), were assumed to occur in only a few symbiotic *Gammaproteobacteria*. We discovered, using comparative genomics, that these rTCA cycle enzymes also occur in some *Chromatiaceae*, including the cultivated sulfur oxidizer Thioflavicoccus mobilis and a gammaproteobacterial metagenome-assembled genome (MAG) from a subsurface aquifer (*Gammaproteobacterium* RIFOXYD12_FULL_61_37) (20) (Fig. 1). The ACLs of tubeworm symbionts and T. mobilis were likely acquired via horizontal gene transfer from other bacterial clades, because the phylogeny of their *aclA* genes is not congruent with their placement in a phylogenomic tree (Fig. 1; see also Fig. S1 in the supplemental material) (16). The tubeworm symbionts and *Thioflavicoccus* also encode either form I or II RuBisCO or both (Text S1, Note 1).

10.1128/mSphere.00394-18.1 TEXT S1 Supplemental methods, notes, and references. Download Text S1, DOCX file, 0.01 MB. Copyright © 2019 Rubin-Blum et al. 2019 Rubin-Blum et al. This content is distributed under the terms of the Creative Commons Attribution 4.0 International license.

10.1128/mSphere.00394-18.2 FIG S1 Phylogeny of ATP-citrate lyase large subunit (AclA). The evolutionary history was inferred by using the maximum likelihood method based on the Le-Gascuel 2008 model (supplemental reference 31). A discrete gamma distribution was used to model evolutionary rate differences among sites (5 categories [+G, parameter = 1.16]). The rate variation model allowed for some sites to be evolutionarily invariable ([+I], 11.2% sites). The tree is drawn to scale, with branch lengths measured in the number of substitutions per site. We used 46 amino acid sequences for the analysis, including all known *aclA* sequences from *Gammaproteobacteria*. All positions containing gaps and missing data were eliminated. There were a total of 707 positions in the final data set. The gray box frames tubeworm sequences; purple color marks *Chromatiaceae* sequences. * marks the sequence of *Magnetococcus marinus*, in which the function of the ATP citrate lyase was biochemically tested. Download FIG S1, EPS file, 2.3 MB. Copyright © 2019 Rubin-Blum et al. 2019 Rubin-Blum et al. This content is distributed under the terms of the Creative Commons Attribution 4.0 International license.

### Presence of the rTCA and CBB pathways in the genome of a single bacterium.

Due to the fragmented nature of the previously available genomes of tubeworm symbionts, past studies could not determine whether the genes for both pathways are present in a single genome or if the two pathways are distributed in a strain-specific manner, i.e., only one of the two pathways is present in the genome of a single cell (4). Here, we provide two lines of evidence that the two pathways can cooccur in the genome of a single organism. First, sequencing coverage for the genes of both pathways in the E. laminata symbiont was similar to that of single-copy marker genes (Table S1). Since genes that are strain specific are expected to have lower coverage than the rest of the genome (21), the similar coverage of genes encoding the two pathways and single-copy genes suggests that in the E. laminata symbiont both pathways are present in all cells. Second, in the closed genome of the cultured T. mobilis, the genes encoding the rTCA and the CBB cycle cooccur, providing evidence that these genes coexist in a single genome.

10.1128/mSphere.00394-18.9 TABLE S1 Uniform sequencing read coverage of the rTCA and Calvin cycle gene clusters and their expression values. All values are normalized to that of the *atpI* gene (ATP synthase F_0_ sector subunit a). Phage genes are shown as an example of a gene cluster that was present only in a subpopulation of the symbionts. The very low expression values for the strain-specific phage cluster are not shown, as the expression values for strain-specific genes cannot be measured accurately. Download Table S1, DOCX file, 0.02 MB. Copyright © 2019 Rubin-Blum et al. 2019 Rubin-Blum et al. This content is distributed under the terms of the Creative Commons Attribution 4.0 International license.

10.1128/mSphere.00394-18.10 TABLE S2 Accession numbers of genomes used in comparative analyses. Download Table S2, DOCX file, 0.01 MB. Copyright © 2019 Rubin-Blum et al. 2019 Rubin-Blum et al. This content is distributed under the terms of the Creative Commons Attribution 4.0 International license.

**FIG 2:**
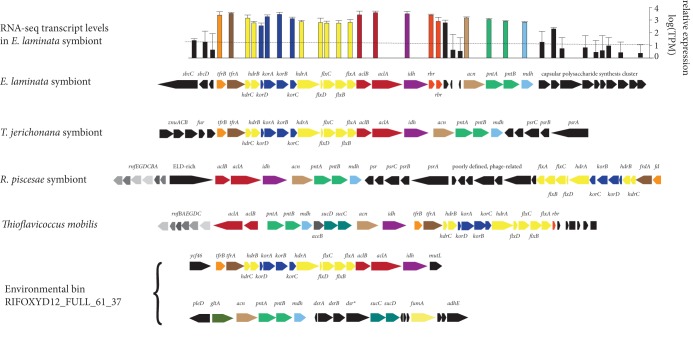
The rTCA cycle gene clusters in symbiotic and free-living bacteria and the respective transcriptomic gene expression levels in the symbionts of Escarpia laminata tubeworm [*aclA*, log(TPM) = 3.6; *korA*, log(TPM) = 3.3; *hdrA*, log(TPM) = 2.9; for comparison, *atpB*, log(TPM) = 2.0; *cbbM*, log(TPM) = 5.0]. TPM, transcripts per kilobase million. *rbr*, rubrerythrin. dsr*, oxidoreductase related to the NADPH-dependent glutamate synthase small chain, clustered with sulfite reductase. The dotted line is the median expression value for E. laminata genes.

Our transcriptomic analyses of E. laminata tubeworm symbionts revealed high expression levels of both the rTCA and the CBB cycle genes (Fig. 2; Table S1). This observation is consistent with previous proteomic analyses of the *Riftia* symbiont (the metabolism of the symbionts from these two tubeworms is highly similar) (6, 7). The high expression levels of genes from the rTCA and the CBB cycle suggest that both pathways play an important metabolic role in these symbionts. It is, however, not clear whether these cycles function simultaneously within single symbiont cells or are differentially expressed within the symbiont population (4).

### The rTCA gene clusters are conserved among the tubeworm symbionts and some *Chromatiaceae* bacteria.

In the tubeworm symbionts, the cultivated T. mobilis, and the gammaproteobacterial MAG from a subsurface aquifer, there was a considerable level of conservation of the rTCA gene clusters, at the sequence and synteny levels (Fig. 2). The *aclAB* genes that encode the two subunits of the ACL were accompanied by those that encode bidirectional TCA cycle enzymes, including *acn* (aconitase), *idh* (isocitrate dehydrogenase), and *mdh* (malate dehydrogenase). The other rTCA-specific genes *korABCD* (four-subunit OGOR) and *tfrAB* (putative thiol:fumarate reductase) were also present in the rTCA gene cluster. Similar to the ACL, the four-subunit OGOR and the thiol:fumarate reductase are very rare among *Gammaproteobacteria* and were probably acquired via a single horizontal gene transfer event from a distant bacterial clade (Fig. 1; Fig. S2 and S3). A dimeric OGOR (*korAB* genes), more common than the four-subunit enzyme among gammaproteobacterial autotrophs, yet absent in T. mobilis, was located elsewhere in the genome of the E. laminata symbiont. The *korAB* genes were colocalized with genes that encode other well-expressed TCA cycle enzymes (Text S1, Note 2; Fig. S4). These well-expressed genes included the citrate synthase (*gltA*) gene, which could indicate its use in the catabolic oxidative TCA cycle. Alternatively, strong expression of citrate synthase could also indicate autotrophic CO_2_ fixation via the recently discovered ACL-independent reverse oxidative TCA (roTCA) cycle (1, 22).

10.1128/mSphere.00394-18.3 FIG S2 Phylogeny of the alpha subunit (KorA) of the 4-subunit 2-oxoglutarate:ferredoxin oxidoreductase (OGOR, 33 amino acid sequences, 362 positions). The evolutionary history was inferred using the maximum likelihood method based on the Le-Gascuel 2008 model (supplemental reference 31). A discrete gamma distribution was used to model evolutionary rate differences among sites (5 categories [+G, parameter = 0.98]). The rate variation model allowed for some sites to be evolutionarily invariable ([+I], 13.0% sites). The tree is drawn to scale, with branch lengths measured in the number of substitutions per site. The gray box frames tubeworm sequences; purple color marks *Chromatiaceae* sequences. Biochemically characterized archaeal sequences were not included in the tree to be able to present the clade of interest in high resolution. Download FIG S2, EPS file, 2.4 MB. Copyright © 2019 Rubin-Blum et al. 2019 Rubin-Blum et al. This content is distributed under the terms of the Creative Commons Attribution 4.0 International license.

10.1128/mSphere.00394-18.4 FIG S3 Phylogeny of the alpha subunit (TfrA) of the thiol:fumarate reductase (21 amino acid sequences, 483 positions). The evolutionary history was inferred using the maximum likelihood method based on the Le-Gascuel 2008 model (supplemental reference 31). A discrete gamma distribution was used to model evolutionary rate differences among sites (5 categories [+G, parameter = 1.22]). The rate variation model allowed for some sites to be evolutionarily invariable ([+I], 17% sites). The tree is drawn to scale, with branch lengths measured in the number of substitutions per site. The gray box frames tubeworm sequences; purple color marks *Chromatiaceae* sequences. The distant TfrA sequence from Methanobacterium thermoautotrophicum was not included in the tree to present the clade of interest in high resolution. Download FIG S3, EPS file, 2.1 MB. Copyright © 2019 Rubin-Blum et al. 2019 Rubin-Blum et al. This content is distributed under the terms of the Creative Commons Attribution 4.0 International license.

10.1128/mSphere.00394-18.5 FIG S4 Genome-based reconstruction of the TCA cycle in tubeworm symbionts. (A) Expression profile of both gene clusters that encode the TCA cycle in *Escarpia laminata* tubeworm symbionts. (B) The TCA cycle reconstruction. Each box is named after the respective protein. Frame color corresponds to a gene cluster, and box color corresponds to the gene color code in panel A. FumB is not colored as the standalone *fumB* gene is located elsewhere in the genome. OAA, oxaloacetate. Download FIG S4, EPS file, 2.5 MB. Copyright © 2019 Rubin-Blum et al. 2019 Rubin-Blum et al. This content is distributed under the terms of the Creative Commons Attribution 4.0 International license.

An array of genes that encode several electron-translocating complexes were integrated into the rTCA cycle gene clusters. These complexes included an electron-bifurcating NADH dehydrogenase/heterodisulfide reductase complex (*flxABCD-hdrABC* genes [Text S1, Note 3] [23]), an NAD(P)^+^ transhydrogenase (24) and Na^+^-translocating Rnf membrane complex (*pntAB* and *rnfABCDGE* genes [Text S1, Note 4] [25]). Most interestingly, the conserved interspersing of the *korABCD* and *tfrAB* genes with the *flxABCD-hdrABC* genes hints at the possibility that these proteins form a complex that efficiently shuttles electrons directly to the OGOR and the thiol:fumarate reductase (Text S1, Note 3, and Fig. S5). If this is the case, the carbon fixation efficiency of the rTCA cycle would be most likely considerably higher than the canonical rTCA cycle.

10.1128/mSphere.00394-18.6 FIG S5 Proposed function of the putative HdrABC-FlxABCD-KorABCD-TfrAB complex. Electrons from NADH oxidation are transferred to HdrA, which may bifurcate them to KorABCD (alternatively mediate electron transfer via ferredoxin) and to TfrAB (alternatively mediate electron transfer via the thiol/disulfide pair of DsrC). Squares and hexagons mark the estimated presence of [4Fe-4S]^2+/1+^ and [2Fe-2S]^2+/1+^ centers, respectively. Download FIG S5, EPS file, 2.2 MB. Copyright © 2019 Rubin-Blum et al. 2019 Rubin-Blum et al. This content is distributed under the terms of the Creative Commons Attribution 4.0 International license.

### Conclusions.

Until now, the only bacteria known to possess both the CBB and rTCA pathways were sulfur-oxidizing, tubeworm symbionts, and possibly also large sulfur bacteria, all of which are currently not amenable to cultivation-based studies. Experimental studies are now feasible in the cultivable T. mobilis, in which the genes for the CBB and rTCA cycles coexist. Such studies would reveal if these pathways are expressed under different physicochemical conditions and potentially allow the biotechnological optimization of efficiency and yield in production processes that rely on autotrophic carbon fixers. To our knowledge, the use of organisms with multiple carbon fixation pathways has not been used as a design principle for these applications.

### Methods. (i) Comparative genomics and transcriptomics.

Publicly available genomes from the NCBI and JGI-IMG collections, as well as *de novo*-assembled genomes of Escarpia laminata symbionts (estimated completeness 99.5%), were used for genomic comparison (see Text S1). To verify presence/absence of target gene homologs in sequenced organisms, we used NCBI’s BLAST against the nucleotide collection and nonredundant protein database (26). E. laminata symbiont genomes were used as a template for genome-centered transcriptomics.

### (ii) Phylogenetic and phylogenomic analyses.

Phylogenomic treeing was performed using scripts available at phylogenomics-tools (https://doi.org/10.5281/zenodo.46122). Twenty-three marker proteins that are universally conserved across the bacterial domain were extracted from genomes using the AMPHORA2 pipeline (27). Twenty-three single-copy markers were used for alignment with MUSCLE (28). The marker alignments were concatenated into a single partitioned alignment, and poorly aligned regions were removed. Functional protein sequences were aligned with MAFFT (29). Maximum likelihood trees were calculated with IQ-TREE (30) and MEGA7 (31), using the best-fitting model.

### Data availability.

Sequences are available under the BioProject accession number PRJNA471406.

10.1128/mSphere.00394-18.7 FIG S6 Phylogeny of the alpha subunit (KorA) from the 2-subunit 2-oxoglutarate:ferredoxin oxidoreductase (OGOR, 44 amino acid sequences, 541 positions). The evolutionary history was inferred by using the maximum likelihood method based on the Le-Gascuel 2008 model (supplemental reference 31). A discrete gamma distribution was used to model evolutionary rate differences among sites (5 categories [+G, parameter = 1.2]). The rate variation model allowed for some sites to be evolutionarily invariable ([+I], 6.0% sites). The tree is drawn to scale, with branch lengths measured in the number of substitutions per site. The gray box frames tubeworm sequences; purple color marks *Chromatiaceae* sequences. * marks the sequence of Mycobacterium tuberculosis, in which the function of the 2-subunit 2-oxoglutarate:ferredoxin oxidoreductase was biochemically tested. Download FIG S6, EPS file, 2.6 MB. Copyright © 2019 Rubin-Blum et al. 2019 Rubin-Blum et al. This content is distributed under the terms of the Creative Commons Attribution 4.0 International license.

10.1128/mSphere.00394-18.8 FIG S7 Phylogeny of the alpha subunit (HdrA) from the NADH dehydrogenase/heterodisulfide reductase (HdrABC-FlxABCD) electron-bifurcating complex, colocalized with the 4-subunit OGOR (38 amino acid sequences, 576 positions). The evolutionary history was inferred by using the maximum likelihood method based on the Le-Gascuel 2008 model (supplemental reference 31). A discrete gamma distribution was used to model evolutionary rate differences among sites (5 categories [+G, parameter = 0.95]). The rate variation model allowed for some sites to be evolutionarily invariable ([+I], 9.3% sites). The tree is drawn to scale, with branch lengths measured in the number of substitutions per site. The gray box frames tubeworm sequences; purple color marks *Chromatiaceae* sequences. *, the function of the HdrABC-FlxABCD was tested in the deltaproteobacterium Desulfovibrio vulgaris. Download FIG S7, EPS file, 2.5 MB. Copyright © 2019 Rubin-Blum et al. 2019 Rubin-Blum et al. This content is distributed under the terms of the Creative Commons Attribution 4.0 International license.
