# Supplementary material for: Genetic Evidence for Two Carbon Fixation Pathways (the Calvin-Benson-Bassham Cycle and the Reverse Tricarboxylic Acid Cycle) in Symbiotic and Free-Living Bacteria
Source: mSphere. 2019 Jan 2;4(1):e00394-18. doi: 10.1128/mSphere.00394-18 (PMC6315080; doi:10.1128/mSphere.00394-18)
Supplement: TEXT S1 [file sph001192735s1.docx]

**Supplemental Text S1 for**

**Genetic evidence for two carbon fixation pathways in symbiotic and free-living bacteria: The Calvin-Bassham cycle and the reverse tricarboxylic acid cycle**

Maxim Rubin-Blum^1,2*^, Nicole Dubilier^1,3^, Manuel Kleiner^4*^

^1^Max-Planck Institute for Marine Microbiology, Celsiusstrasse 1, 28359 Bremen, Germany

^2^Israel Limnology and Oceanography Research, Tel Shikmona, 3108000, Haifa, Israel

^3^MARUM, University of Bremen, 28359 Bremen, Germany

^4^Department of Plant & Microbial Biology, North Carolina State University, Raleigh, NC, USA

*Corresponding authors

mrubin@ocean.org.il

manuel_kleiner@ncsu.edu

**Contents:**

1. Supplemental Methods
2. Supplemental Notes

**Supplemental Methods**

**Tubeworm collection**: Three *Escarpia laminata* individuals were collected with a remotely-operated vehicle (ROV) MARUM-Quest 4000 m during the RV Meteor M114-2 cruise to the Campeche Knolls in March 2015 (21°54' N; 93°26' W, water depth of 3100 m). The collection site is described in detail in another publication (1). Seven trophosome subsamples from three individuals were fixed in RNAlater (Sigma, Steinheim, Germany) for transcriptomic and metagenomic analyses according to the manufacturer’s instructions upon recovery and stored at -80 °C until processed.

**DNA and RNA extraction and sequencing**: We extracted DNA and RNA from 4 subsamples of a single tubeworm trophosome. DNA and RNA were extracted in parallel with the AllPrep DNA/RNA Mini Kit (Qiagen, Hilden, Germany) following the standard protocol. DNA/RNA quality was assessed with the Agilent 2100 Bioanalyzer. (Agilent, Santa Clara, USA). Ovation RNA-seq System V2 (NuGen, San Carlos, CA, USA) was used to synthesize cDNA. Genomic DNA and cDNA libraries were generated with the DNA library prep kit for Illumina (BioLABS, Frankfurt am Main, Germany). 15-18 genomic DNA and 11-64 million complementary DNA 150 bp paired-end reads were sequenced for each subsample on the Illumina HiSeq 2500 platform at the Max Planck Genome Centre (Cologne).

**Metagenomics**: Individual metagenomes were assembled with IDBA-UD (2) or with SPAdes V3.9 in metaSPAdes mode (3, 4), following decontamination, quality filtering (QV=2) and adapter trimming of the reads with the BBDuk tool from the BBMap suite (Bushnell B, <http://sourceforge.net/projects/bbmap/>). The symbiont genomes were binned based on genome coverage, GC content, taxonomic affiliation and the differential coverage using gbtools (5, 6). We reassembled the genomes with SPAdes V3.9, using a maximum k-mer length of 127, following re-mapping of Illumina reads to bins using BBMap with 0.98 minimum identity. Alternatively, we explored assembly graphs produced by metaSPAdes with Bandage software (7) and collected nodes connected to the *E. laminata* SOX 16S rRNA gene sequences. Both methods produced similar bins. Quality metrics were calculated with Quast (8) and CheckM (9). The genomes were annotated with RAST (10) and the DOE-JGI Microbial Genome Annotation Pipeline (11). The annotations were manually cross-checked and the annotations for the genes discussed here were verified using NCBI’s BLAST (12). Iron sulfur cluster binding motifs were determined with MOTIF search against the Pfam database (13, 14).

***Transcriptome analysis***

Adapters and symbiont ribosomal genes were removed from transcriptome reads with BBDuk. Transcriptome reads from each individual were mapped to assembled symbiont genomes with BBMap using a minimum identity value of 0.98. Mapped reads were assigned to genomic features with featureCounts. The read counts were normalized within individual samples with the transcripts per million calculation (TPM), (15).

**Supplemental Notes**

**Supplemental Note 1**: **Ribulose-1,5-bisphosphate carboxylase/oxygenase (RuBisCO) forms in tubeworm symbionts and related bacteria**: The genomes of the tubeworm symbionts encode a single Form II RuBisCO (*cbbM* gene). In contrast, the photolithoautotrophic Chromatiaceae, *Thioflavicoccus mobilis* in particular, encode both the Forms II and I RuBisCO. *T. mobilis* encode two copies of Form I RuBisCO, both common among Chromatiaceae: the proteobacterial type IAq, as well as the cyanobacterial type IBc, which is co-localized with a set of genes that encode a carboxisome, a protein complex that concentrates carbon dioxide to increase the efficiency of RuBisCO (16). Two forms of RuBisCo also occasionaly occur among chemolithoautotrophic symbiotic bacteria, for example the symbionts of the scaly snail *Chrysomallon squamiferum* encode both IAq and type II forms of RuBisCo (17). Beggiatoaceae appear to encode either type I or type II RuBisCO. In summary, carbon fixation via the CBB cycle is widespread among the gammaproteobacterial autotrophs.

**Supplemental Note 2**: **The function of the dimeric KorAB in tubeworm symbionts**: : Two forms of the 2-oxoglutarate:ferredoxin oxidoreductase, the four-subunit KorABCD and the two subunit KorAB, were encoded in genomes of *Escarpia*, *Riftia*, *Tevnia* and *Ridgeia* tubeworm symbionts, as well as in the environmental metagenomic bin (gammaproteobacterium RIFOXYD12_FULL_61_37, assembly MGZB00000000.1). Similar to KorABCD, the dimeric 2-oxoglutarate:ferredoxin oxidoreductase (OGOR) may catalyze the reductive carboxylation of succinyl-CoA, as it has been shown in the anaerobic bacterium *Hydrogenobacter thermophilus*(18).

In tubeworm symbionts, the *korAB* genes (the dimeric 2-oxoglutarate:ferredoxin oxidoreductase) were co-localized with genes that encode additional bidirectional TCA cycle enzymes (**Suppl. Fig. S4**). Besides the *korAB*, this second cluster of TCA cycle genes in the *E. laminata* symbiont included those that encode a pyruvate:ferredoxin oxidoreductase (PFOR), (**Suppl. Fig. S4**). Co-occurrence of genes that encode OGOR and PFOR is not surprising, as the reductive carboxylation of acetyl-CoA into pyruvate via PFOR allows for carbon flow into other central metabolites and thus PFOR plays an important role in bacteria that use the reverse TCA cycle to assimilate carbon (19, 20). Together with a standalone *fumB* gene (fumarate hydratase), these two clusters comprise the set of genes needed for the complete rTCA cycle (**Suppl. Fig. S4**).

The dimeric korAB is likely involved not only in the reductive, but also in the oxidative TCA cycle. The sequences of the *korAB* genes resembled those of *Cand*. Maribeggiatoa from the Guaymas Basin (70 and 65 % similarity of *korA* and *korB* at amino acid level, respectively). In *Cand*. Maribeggiatoa, as well as in *Mycobacterium tuberculosis*, this dimer has been suggested to fulfill the function of the oxidative 2-oxoglutarate dehydrogenase complex (21, 22). Thus, the dimeric OGOR may function in both the oxidative and the reductive directions of the TCA cycle. Given that the symbionts of tubeworms store energy-rich glycogen (23), a functional oxidative TCA cycle is needed to use this storage during periods of energy deprivation. Interestingly, the genomes of the tubeworm symbionts appear to lack the *ogdh* gene that encodes the oxoglutarate decarboxylase subunit of the oxoglutarate dehydrogenase complex, thus the oxidative decarboxylation of 2-oxoglutarate and the functionality of the oxidative TCA cycle may depend on the reversibility of the dimeric OGOR. The fact that in *Escarpia laminata* symbionts the *korA* and *korB* genes are co-localized with *gltA* (citrate synthase, which is required for the oxidative function of the TCA cycle), albeit in opposing directions (**Suppl. Fig. S4**), provides further evidence for a role of the dimeric OGOR in the oxidative TCA cycle.

Interestingly, the dimeric OGOR is more common in Gamaproteobacteria than the four-subunit one, and the two versions co-occur primarily in the tubeworm symbiont (**Main text Figure 1**, **Suppl. Fig. S6**). Both the dimeric and the four-subunit OGORs were expressed by the tubeworm symbionts (**Suppl. Fig. S4**), and both appear to be encoded in each individual genome based on coverage (**Suppl. Table 1**). This provides an opportunity to look into their function in the future.

**Supplemental Note 3**: **Evidence for potential involvement of FlxABCD-HdrABC** in **electron flow to the rTCA cycle:** Interestingly, the *korABCD* and *tfrAB* genes in all Gammaproteobacteria with the rTCA cycle are co-localized in an operon-like arrangement with genes that are homologous to the flavin-based, electron-bifurcating NADH dehydrogenase/heterodisulfide reductase complex (FlxABCD-HdrABC, **Suppl. Fig. S4**). FlxABCD-HdrABC proteins are widespread among several bacterial classes and have been studied primarily in deltaproteobacterial anaerobic sulfate-reducers, in which they conserve energy by bifurcating electrons from NADH to ferredoxin and dithiol/disulfide redox pair of DsrC protein (24–26). In these Deltaproteobacteria, the *flxABCD-hdrABC* cluster is co-localized and co-transcribed with an alcohol dehydrogenase, which catalyzes ethanol oxidation/pyruvate fermentation and creates electron flow between NADH, ferredoxin and DsrC (25). We have not found genes encoding an ethanol dehydrogenase in the gammaproteobacterial genomes with the rTCA cycle gene cluster. The fact that *flxABCD*-*hdrABC*, *tfrAB* and *korABCD* genes are co-localized with same directionality and share evolutionary history (**Suppl. Fig. S2, S3 and S7**), hints at the functional alliance between the respective proteins. This is not surprising, given the ability of the FlxABCD-HdrABC complex to reduce ferredoxin needed for fixation of CO_2_ via the 2-oxoglutarate:ferredoxin oxidoreductase (27). Moreover, reduction of heterosulfide via the heterodisulfide reductase complex to thiol may fuel the thiol-dependent reduction of fumarate to succinate via the thiol:fumarate reductase. Most intriguingly, the conserved interspersing of the *korABCD* genes with the *hdrABC* and *tfrAB* genes hints at the possibility that these proteins form a novel complex that bifurcates electrons directly to OGOR and thiol:fumarate reductase (**Suppl. Fig. S5**). We expect the direct electron shuttling via flavin-based bifurcation to be highly efficient in comparison to the indirect electron shuttling via ferredoxin and thiol/disulfide (28).

**Supplemental Note 4**: **NAD(P)+ transhydrogenases and Rnf complexes may facilitate electron flow to the rTCA cycle:** Apart from the FlxABCD-HdrABC proteins, NAD(P)+ transhydrogenases (*pntAB* genes) and Na+ translocating Rnf membrane complexes (*rnfABCDGE* genes) may facilitate electron flow to the rTCA cycle. Similar to the *flxABCD-hdrABC* gene cluster, the *pntAB* and *rnfABCDGE* genes were found in the genomic cluster with the rTCA/TCA cycle genes (**Suppl. Fig. S4**).

NAD(P)^+^ transhydrogenases use proton motive force to catalyze electron transfer between NADH and NADPH (29). Since under most physiological conditions formation of NADPH is favorable (29), NAD(P)^+^ transhydrogenase may couple the proton motive force to NADPH-dependent reductive carboxylation of α-ketoglutarate via isocitrate dehydrogenase (**Suppl. Fig. S4**).

Na+ translocating Rnf membrane complex is known to mediate electron flow between reduced pyridine nucleotides to the cellular ferredoxin pool, under both anoxic and oxic conditions (30). Recycling of reduced ferredoxin is beneficial for the ferredoxin dependent carboxylases, OGOR and PFOR. Indeed, two *rnfABCDGE* gene clusters were colocalized and cotranscribed with the two copies of well-transcribed PFOR-encoding *por* genes from genomes of the vestimentiferan symbionts, suggesting dependence of PFORs on ferredoxin recycling via the Rnf complexes (**Suppl. Fig. S4**).

**References:**

1. Sahling H, Borowski C, Escobar-Briones E, Gaytán-Caballero A, Hsu C-W, Loher M, MacDonald I, Marcon Y, Pape T, Römer M, Rubin-Blum M, Schubotz F, Smrzka D, Wegener G, Bohrmann G. 2016. Massive asphalt deposits, oil seepage, and gas venting support abundant chemosynthetic communities at the Campeche Knolls, southern Gulf of Mexico. Biogeosciences 13:4491–4512.

2. Peng Y, Leung HCM, Yiu SM, Chin FYL. 2012. IDBA-UD: a de novo assembler for single-cell and metagenomic sequencing data with highly uneven depth. Bioinformatics 28:1420–8.

3. Nurk S, Bankevich A, Antipov D. 2013. Assembling genomes and mini-metagenomes from highly chimeric reads. Res Comput Mol Biol 158–170.

4. Bankevich A, Nurk S, Antipov D, Gurevich AA., Dvorkin M, Kulikov AS, Lesin VM, Nikolenko SI, Pham S, Prjibelski AD, Pyshkin AV., Sirotkin AV., Vyahhi N, Tesler G, Alekseyev MA, Pevzner PA. 2012. SPAdes: A new genome assembly algorithm and its applications to single-cell sequencing. J Comput Biol 19:455–477.

5. Albertsen M, Hugenholtz P, Skarshewski A, Nielsen KL, Tyson GW, Nielsen PH. 2013. Genome sequences of rare, uncultured bacteria obtained by differential coverage binning of multiple metagenomes. Nat Biotechnol 31:533–538.

6. Seah BKB, Gruber-Vodicka HR. 2015. gbtools: Interactive visualization of metagenome bins in R. Front Microbiol 6:1451.

7. Wick RR, Schultz MB, Zobel J, Holt KE. 2015. Bandage: Interactive visualization of de novo genome assemblies. Bioinformatics 31:3350–3352.

8. Gurevich A, Saveliev V, Vyahhi N, Tesler G. 2013. QUAST: quality assessment tool for genome assemblies. Bioinformatics 29:1072–5.

9. Parks DH, Imelfort M, Skennerton CT, Hugenholtz P, Tyson GW. 2015. CheckM : assessing the quality of microbial genomes recovered from isolates , single cells , and metagenomes. Genome Res 25:1043–1055.

10. Overbeek R, Olson R, Pusch GD, Olsen GJ, Davis JJ, Disz T, Edwards RA, Gerdes S, Parrello B, Shukla M, Vonstein V, Wattam AR, Xia F, Stevens R. 2014. The SEED and the Rapid Annotation of microbial genomes using Subsystems Technology (RAST). Nucleic Acids Res 42:D206–D214.

11. Huntemann M, Ivanova NN, Mavromatis K, Tripp HJ, Paez-Espino D, Palaniappan K, Szeto E, Pillay M, Chen I-MA, Pati A, Nielsen T, Markowitz VM, Kyrpides NC. 2015. The standard operating procedure of the DOE-JGI Microbial Genome Annotation Pipeline (MGAP v.4). Stand Genomic Sci 10:86.

12. Johnson M, Zaretskaya I, Raytselis Y, Merezhuk Y, McGinnis S, Madden TL. 2008. NCBI BLAST: a better web interface. Nucleic Acids Res 36:W5–W9.

13. Finn RD, Bateman A, Clements J, Coggill P, Eberhardt RY, Eddy SR, Heger A, Hetherington K, Holm L, Mistry J, Sonnhammer ELL, Tate J, Punta M. 2014. Pfam: The protein families database. Nucleic Acids Res 42:222–230.

14. MOTIF Search, http://www.genome.jp/tools/motif/

15. Wagner GP, Kin K, Lynch VJ. 2012. Measurement of mRNA abundance using RNA-seq data: RPKM measure is inconsistent among samples. Theory Biosci 131:281–285.

16. Badger MR, Bek EJ. 2008. Multiple Rubisco forms in proteobacteria: Their functional significance in relation to CO_2_ acquisition by the CBB cycle. J Exp Bot 59:1525–1541.

17. Nakagawa S, Shimamura S, Takaki Y, Suzuki Y, Murakami S, Watanabe T, Fujiyoshi S, Mino S, Sawabe T, Maeda T, Makita H, Nemoto S, Nishimura S-I, Watanabe H, Watsuji T, Takai K. 2014. Allying with armored snails: the complete genome of gammaproteobacterial endosymbiont. ISME J 8:40–51.

18. Yamamoto M, Arai H, Ishii M, Igarashi Y. 2006. Role of two 2-oxoglutarate:ferredoxin oxidoreductases in *Hydrogenobacter thermophilus* under aerobic and anaerobic conditions. FEMS Microbiol Lett 263:189–193.

19. Evans M, Buchanan B, Arnon D. 1966. A new ferredoxin-dependent carbon reduction cycle in a photosynthetic bacterium. Proc Natl Acad Sci 55:928–934.

20. Hügler M, Wirsen CO, Fuchs G, Taylor CD, Sievert SM, Hügler M, Craig D. 2005. Hügler M, Wirsen CO, Fuchs G, Taylor CD, Sievert SM, Hügler M, Craig D. 2005. Evidence for autotrophic CO_2_ fixation via the reductive rricarboxylic acid cycle by members of the ε subdivision of proteobacteria. J Bacteriol 187:3020–3027.

21. Baughn AD, Garforth SJ, Vilchèze C, Jacobs WR. 2009. An anaerobic-type α-ketoglutarate ferredoxin oxidoreductase completes the oxidative tricarboxylic acid cycle of *Mycobacterium tuberculosis*. PLoS Pathog 5:1–10.

22. MacGregor BJ, Biddle JF, Harbort C, Matthysse AG, Teske A. 2013. Sulfide oxidation, nitrate respiration, carbon acquisition, and electron transport pathways suggested by the draft genome of a single orange Guaymas Basin Beggiatoa (*Cand*. Maribeggiatoa) sp. filament. Mar Genomics 11:53–65.

23. Sorgo A, Gaill F, Lechaire JP, Arndt C, Bright M. 2002. Glycogen storage in the *Riftia pachyptila* trophosome: Contribution of host and symbionts. Mar Ecol Prog Ser 231:115–120.

24. Pereira IAC, Ramos AR, Grein F, Marques MC, da Silva SM, Venceslau SS. 2011. A comparative genomic analysis of energy metabolism in sulfate reducing bacteria and archaea. Front Microbiol 2:1–22.

25. Ramos AR, Grein F, Oliveira GP, Venceslau SS, Keller KL, Wall JD, Pereira IAC. 2015. The FlxABCD-HdrABC proteins correspond to a novel NADH dehydrogenase/heterodisulfide reductase widespread in anaerobic bacteria and involved in ethanol metabolism in *Desulfovibrio vulgaris* Hildenborough. Environ Microbiol 17:2288–2305.

26. Meyer B, Kuehl J V., Price MN, Ray J, Deutschbauer AM, Arkin AP, Stahl DA. 2014. The energy-conserving electron transfer system used by *Desulfovibrio alaskensis* strain G20 during pyruvate fermentation involves reduction of endogenously formed fumarate and cytoplasmic and membrane-bound complexes, Hdr-Flox and Rnf. Environ Microbiol 16:3463–3486.

27. Erb TJ. 2011. Carboxylases in natural and synthetic microbial pathways. Appl Environ Microbiol 77:8466–8477.

28. Buckel W, Thauer RK. 2018. Flavin-based electron bifurcation, a new mechanism of biological energycoupling. Chem Rev 118:3862–3886.

29. Spaans SK, Weusthuis RA, van der Oost J, Kengen SWM. 2015. NADPH-generating systems in bacteria and archaea. Front Microbiol 6:1–27.

30. Biegel E, Schmidt S, González JM, Müller V. 2011. Biochemistry, evolution and physiological function of the Rnf complex, a novel ion-motive electron transport complex in prokaryotes. Cell Mol Life Sci 68:613–634.

31. Le SQ, Gascuel O. 2008. An improved general amino acid replacement matrix. Mol Biol Evol 25:1307–1320.
