# Supplementary material for: Genetic Evidence for Two Carbon Fixation Pathways (the Calvin-Benson-Bassham Cycle and the Reverse Tricarboxylic Acid Cycle) in Symbiotic and Free-Living Bacteria
Source: mSphere. 2019 Jan 2;4(1):e00394-18. doi: 10.1128/mSphere.00394-18 (PMC6315080; doi:10.1128/mSphere.00394-18)
Supplement: TABLE S1 [file sph001192735st1.docx]

**Supplemental Table S1**

| Gene | Coverage normalized to gene length and *atpI* | SD (n=7) | Expression (log[TPM+1]) | SD (n=4) |
| --- | --- | --- | --- | --- |
| **ATP synthase** |  |  |  |  |
| *atpI* | 1.00 | 0.00 | 1.96 | 0.31 |
| *atpE* | 0.95 | 0.13 | 2.85 | 0.04 |
| *atpF* | 1.02 | 0.11 | 2.61 | 0.19 |
| *atpD* | 0.87 | 0.08 | 1.80 | 1.25 |
| *atpA* | 1.05 | 0.11 | 2.70 | 0.22 |
| *atpG* | 1.09 | 0.08 | 2.51 | 0.22 |
| *atpB* | 1.11 | 0.10 | 2.93 | 0.05 |
| *atpC* | 1.06 | 0.09 | 2.92 | 0.23 |
| **rTCA cycle cluster 1** |  |  |  |  |
| putative *frdB* | 1.11 | 0.18 | 3.38 | 0.26 |
| putative *frdA* | 1.01 | 0.16 | 3.51 | 0.10 |
| *hdrC* | 0.92 | 0.11 | 3.15 | 0.24 |
| *hdrB* | 0.91 | 0.15 | 2.77 | 0.14 |
| *korD* | 0.51 | 0.11 | 2.53 | 0.22 |
| *korA* | 0.97 | 0.13 | 3.26 | 0.14 |
| *korB* | 1.19 | 0.20 | 3.43 | 0.17 |
| *korC* | 1.21 | 0.21 | 3.11 | 0.12 |
| *hdrA* | 1.14 | 0.13 | 2.86 | 0.11 |
| *flxD* | 1.16 | 0.15 | 2.80 | 0.35 |
| *flxC* | 0.91 | 0.15 | 2.75 | 0.16 |
| *flxB* | 1.04 | 0.17 | 2.75 | 0.21 |
| *flxA* | 1.04 | 0.08 | 2.82 | 0.22 |
| *aclB* | 1.18 | 0.12 | 3.41 | 0.30 |
| *aclA* | 1.11 | 0.15 | 3.58 | 0.12 |
| *idh* | 1.07 | 0.09 | 3.49 | 0.20 |
| Rubrerythrin | 1.01 | 0.09 | 3.38 | 0.07 |
| Rubrerythrin | 1.15 | 0.06 | 2.88 | 0.31 |
| **rTCA cycle cluster 2** |  |  |  |  |
| *sucD* | 1.44 | 0.26 | 3.10 | 0.05 |
| *sucC* | 1.27 | 0.19 | 2.83 | 0.16 |
| *por* | 1.27 | 0.23 | 2.37 | 0.24 |
| *rnfB* | 1.05 | 0.16 | 1.65 | 1.12 |
| *por-like* | 1.25 | 0.19 | 2.64 | 0.26 |
| *korB* | 1.11 | 0.19 | 2.98 | 0.11 |
| *korA* | 1.24 | 0.14 | 2.70 | 0.09 |
| *nuoF** | 1.18 | 0.15 | 1.95 | 0.34 |
| *frdx** | 1.22 | 0.17 | 1.37 | 0.91 |
| ferredoxin | 1.16 | 0.12 | 0.78 | 0.90 |
| *gltA* | 1.18 | 0.09 | 2.75 | 0.06 |
| **Calvin cycle cluster** |  |  |  |  |
| *prkB* | 1.25 | 0.10 | 2.67 | 0.05 |
| *gloB* | 1.16 | 0.13 | 1.17 | 0.78 |
| *dxs* | 1.14 | 0.10 | 1.55 | 0.27 |
| *cbbr* | 1.19 | 0.09 | 0.87 | 1.02 |
| FOG: GGDEF domain | 1.25 | 0.12 | 1.53 | 1.04 |
| *cbbM* | 1.14 | 0.11 | 4.97 | 0.09 |
| hypothetical protein | 1.20 | 0.24 | 2.12 | 0.45 |
| COG1272: Predicted membrane protein hemolysin III homolog gene | 1.19 | 0.09 | 1.87 | 0.33 |
| *cbbQ* | 1.42 | 0.19 | 2.17 | 0.32 |
| *cbbO* | 1.25 | 0.15 | 2.09 | 0.05 |
| *kdph* | 1.24 | 0.15 | 1.93 | 0.20 |
| **Dsr cluster** |  |  |  |  |
| *dsrR* | 1.20 | 0.12 | 1.37 | 0.94 |
| Cobyrinic acid A, CC-diamide synthase | 0.84 | 0.10 | 1.13 | 0.77 |
| *dsrP* | 1.06 | 0.07 | 2.79 | 0.16 |
| *dsrO* | 1.05 | 0.06 | 2.93 | 0.15 |
| *dsrJ* | 1.02 | 0.05 | 2.88 | 0.12 |
| Protein similar to glutamate synthase [NADPH] small chain, clustered with sulfite reductase | 0.88 | 0.11 | 3.29 | 0.05 |
| *dsrK* | 1.14 | 0.14 | 2.66 | 0.15 |
| *dsrM* | 1.08 | 0.09 | 2.58 | 0.16 |
| *tusA* | 1.08 | 0.14 | 3.42 | 0.08 |
| *tusB* | 1.01 | 0.08 | 3.31 | 0.09 |
| *tusC* | 0.83 | 0.06 | 3.48 | 0.10 |
| *tusD* | 0.84 | 0.07 | 3.29 | 0.13 |
| *dsrB* | 0.88 | 0.06 | 3.48 | 0.09 |
| *dsrA* | 1.13 | 0.07 | 3.86 | 0.07 |
| **Strain-specific phage cluster*** |  |  |  |  |
| Mobile element protein | 0.13 | 0.11 |  |  |
| hypothetical protein | 0.27 | 0.46 |  |  |
| hypothetical protein | 0.20 | 0.34 |  |  |
| Phage terminase, large subunit | 0.28 | 0.47 |  |  |
| hypothetical protein | 0.28 | 0.48 |  |  |
| hypothetical protein | 0.36 | 0.62 |  |  |
| hypothetical protein | 0.10 | 0.20 |  |  |
| hypothetical protein | 0.29 | 0.50 |  |  |
| Phage antirepressor protein | 0.35 | 0.60 |  |  |
| hypothetical protein | 0.33 | 0.57 |  |  |
| hypothetical protein | 0.37 | 0.63 |  |  |
| hypothetical protein | 0.38 | 0.68 |  |  |
| hypothetical protein | 0.55 | 0.94 |  |  |
| Phage portal protein | 0.36 | 0.61 |  |  |
| Peptidase S49 | 0.31 | 0.54 |  |  |
| hypothetical protein | 0.23 | 0.40 |  |  |
| elements of external origin, phage-related functions and prophages | 0.29 | 0.50 |  |  |
| hypothetical protein | 0.31 | 0.53 |  |  |
| hypothetical protein | 0.25 | 0.43 |  |  |
| hypothetical protein | 0.34 | 0.58 |  |  |
| hypothetical protein | 0.70 | 1.20 |  |  |
| hypothetical protein | 0.31 | 0.53 |  |  |
| hypothetical protein | 0.24 | 0.41 |  |  |
| hypothetical protein | 0.18 | 0.31 |  |  |
| hypothetical protein | 0.19 | 0.33 |  |  |
| Phage protein | 0.23 | 0.39 |  |  |
| hypothetical protein | 0.07 | 0.11 |  |  |
| Phage protein | 0.27 | 0.49 |  |  |
| Phage protein | 0.22 | 0.38 |  |  |
| hypothetical protein | 0.09 | 0.16 |  |  |
| hypothetical protein | 0.24 | 0.40 |  |  |
| hypothetical protein | 0.31 | 0.54 |  |  |
| Gene Transfer Agent (GTA) ORFG12 | 0.30 | 0.53 |  |  |
| Gene Transfer Agent FAD/FMN-containing dehydrogenase | 0.26 | 0.45 |  |  |
| NLP/P60 family protein | 0.22 | 0.38 |  |  |
| *livF* | 0.28 | 0.48 |  |  |
| hypothetical protein | 0.28 | 0.50 |  |  |
| hypothetical protein | 0.32 | 0.55 |  |  |
| hypothetical protein | 0.47 | 0.82 |  |  |
