# Supplementary material for: Genetic Evidence for Two Carbon Fixation Pathways (the Calvin-Benson-Bassham Cycle and the Reverse Tricarboxylic Acid Cycle) in Symbiotic and Free-Living Bacteria
Source: mSphere. 2019 Jan 2;4(1):e00394-18. doi: 10.1128/mSphere.00394-18 (PMC6315080; doi:10.1128/mSphere.00394-18)
Supplement: TABLE S2 [file sph001192735st2.docx]

**Supplemental Table S2**

| **Genome** | **Accession** |
| --- | --- |
| Endosymbiont of *Escarpia laminata* | BioProject PRJNA471406 |
| Endosymbiont of *Ridgeia piscesae* | GCA_001443675.1 |
| Endosymbiont of *Riftia pachyptila* | GCA_000224455.2 |
| Endosymbiont of Tevnia jerichonana | GCA_000224925.2 |
| Endosymbiont of *Codakia orbicularis* | GCA_001715975.1 |
| Endosymbiont of *Loripes lucinalis* | GCA_001708965.1 |
| Endosymbiont of *Lamellibrachia* sp.Mid-Cayman Rise | Provided by Reveillaud, J., reads available in BioProject PRJEB19217 |
| Endosymbiont of *Escarpia* sp. Mid-Cayman Rise | Provided by Reveillaud, J., reads available in BioProject PRJEB19217 |
| *Sedimenticola selenatireducens* | GCA_000428045.1 |
| *Sedimenticola thiotaurini* | GCA_001007875.1 |
| Gammaproteobacterium RIFOXYD12_FULL_61_37 | MGZB00000000.1 |
| *Achromatium palustre* | GCA_001044195.1 |
| *Thiohalocapsa* sp. ML1 | GCA_001469165.1 |
| Symbiont of *Olavius algarvensis* | DS021107-196.1 |
| Ectosymbiont of *Laxus oneistus* | GCA_900092655.1 |
| *Thioflavicoccus mobilis* | GCA_000327045.1 |
| *Thiococcus pfennigii* | DOE Joint Genome Institute: Gp0139227 |
| *Lamprocystis purpurea* | GCA_000379525.1 |
| *Marichromatium gracile* | GCA_001583505.1 |
| *Thiocapsa marina* | GCA_000223985.2 |
| *Thiocystis violascens* | GCA_000227745.3 |
| *Allochromatium vinosum* | GCA_000025485.1 |
| *Thiorhodococcus drewsii* | GCA_000224065.2 |
| Endosymbiont of *Solemya velum* | GCA_000787395.1 |
| *Thiolapillus brandeum* | GCA_000828615.1 |
| Endosymbiont of *Crysomallon squamiferum* | GCA_000801295.1 |
| Endosymbiont of *Solemya velesiana* | GCF_002020875.1 |
| *Cand*. Maribeggiatoa sp. Guyamas Basin | DOE Joint Genome Institute: Gi01404 |
| *Thioploca ingrica* | GCA_000828835.1 |
| *Beggiatoa alba* | GCA_000245015.1 |
| *Beggiatoa leptomitiformis* | GCA_001305575.2 |
